# Editorial: Innovative high value-added processing of soybean and its by-products

**DOI:** 10.3389/fnut.2023.1240249

**Published:** 2023-06-27

**Authors:** Bo Lyu, Fengzhong Wang, Yang Li, Siew Young Quek, Hansong Yu

**Affiliations:** ^1^College of Food Science and Engineering, Jilin Agriculture University, Changchun, China; ^2^Soybean Research & Development Center, Division of Soybean Processing, Chinese Agricultural Research System, Changchun, China; ^3^Institute of Food Science and Technology, Chinese Academy of Agricultural Sciences, Beijing, China; ^4^College of Food Science, Northeast Agricultural University, Harbin, China; ^5^School of Chemical Sciences, University of Auckland, Auckland, New Zealand

**Keywords:** soybean, high-value, comprehensive utilization, nutritional composition, by-product

## 1. Introduction

Soybean, as a source of high-quality plant-based protein and oil intake for humans, has gradually gained recognition from most consumers for its nutritional value. However, as a traditional food processing, the soybean processing industry has common problems such as resource waste, environmental pollution, and low added value. How to enhance the nutritional value and added value of soybean based on their excellent nutritional advantages through modern food processing methods has gradually become the main task of the industry at this stage.

In this Research Topic, Kong et al. considered the development and utilization of soybean peptides. Li L.-l. et al., Zhang et al., and Agyenim-Boateng et al. contributed to their work on the evaluation of trace functional factors in soybean. Xu et al. improved the utilization of soybean by-products through microbial fermentation technology. The work of Li Z. et al. focused on the green utilization of soybean oil. Wang et al. paid attention to the use of multi-component to improve the nutritional and processing properties of soybean tissue protein. A total of 7 manuscripts were accepted to this Research Topic from various countries, which were both original research articles. The above content covers the development direction of high-value processing and utilization of soybeans, providing good guidance for scholars. This editorial will reorganize the development prospects of *Innovative high-value-added processing of soybean and its by-products*.

## 2. Soybean processing industry chain

**Figure 1:**
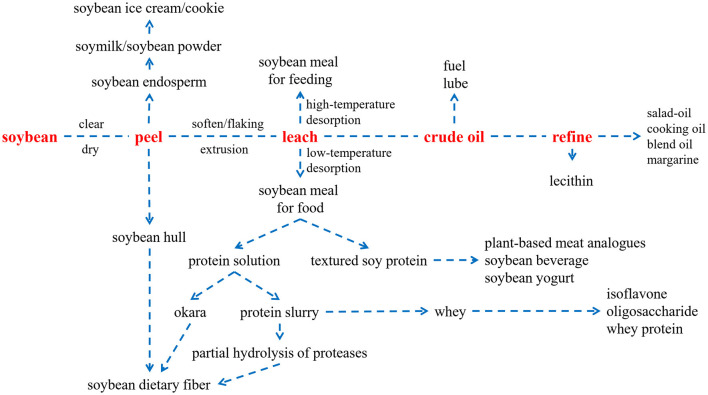
Soybean processing chain (oil as the main chain).

If soybean oil is taken as the main chain, which is the most abundant processed soybean product, the processing chain of soybean can be shown in Figure 1. As shown, in addition to traditional soy products, many refined products have the potential for high-value processing and potential functionality, such as dietary fiber (1), isoflavones (2), soy peptides (3), etc. Therefore, how to accurately analyze the nutritional function of soybean ingredients, so that it is upgraded from ordinary food to functional food, is the most important to achieve the high value of soybean utilization.

## 3. Development prospects

### 3.1. Using special-use soybean varieties for different products to enhance the processing applicability

Varieties have a significant impact on the composition of the main components of soybeans (4, 5), meanwhile, the ingredients have a huge impact on the processing characteristics and nutritional properties of the product, especially the protein (6, 7). However, due to the actual situation of the processing industry, it is difficult to promote special-use soybean varieties, which puts high demands on soybean breeding, that is, both to select and breed high-yielding and adaptable varieties, but also to consider the processing characteristics of varieties and nutritional characteristics to facilitate the promotion of soybean products well high-value. Unfortunately, this work is now only on a small scale and has not been replicated on a large scale.

### 3.2. Deep processing and high-value utilization of soybean by-products represented by soybean dregs (okara) and soybean whey

Soybean whey and okara are typical by-products of traditional soybean products, and although they are often discarded, they are not waste. Soybean whey is rich in proteins, simple sugars, oligosaccharides, minerals, and soy isoflavones (8), meanwhile, okara is a good source of dietary fiber and oligopeptides (3, 9). The above ingredients have been shown to have significant physiological functions, such as immune regulation (10), affect the hormone metabolism of the body (11), weight loss and sugar reduction (12, 13), prevention of colon diseases (14, 15), etc. There are already processing companies that use the above ingredients as raw materials for functional foods, but their market share is small. On the one hand, the cost of deep processing of by-products is too high for general processing enterprises to bear. For example, membrane separation technology, which is considered to be the best way to separate functional components in soybean whey (16), has rarely been applied on a large scale. On the other hand, the detailing of by-product components needs further research. For example, how the residual acidic protein can be separated and utilized, and how the various components in dietary fiber exist. All of the above issues lack systematic research.

### 3.3. Simultaneous production of high-quality oil and physiologically active soy protein using novel processing technologies

In the traditional method, the separation of oil and protein in soybeans is mainly based on pressing and organic solvent extraction. This poses two problems: the residual problem of organic solvents in the oil and the loss of functionality of the protein (17, 18). The optimal solution to this problem is the simultaneous preparation of soy protein and oil using an enzyme-assisted aqueous extraction method, and there have been numerous studies (19, 20). Moreover, the proteins prepared using this method have significant physiological functionality (21). The failure of the enzyme-assisted aqueous extraction method to replace the traditional process for the preparation of soybean oil is mainly attributed to the high cost of the enzymes used and the instability of the conditions after scale-up production. Therefore, there is still a need to strengthen the research of this method in order to realize the industrialization as soon as possible.

### 3.4. Nutritional and functional evaluation of the subdivided components in soybeans

Researchers are still preferring to use the logic of nutrient classification to analyze the functionality of soy components. But in reality, it is the many subdivided components that are functional, such as β-globulin [part of soybean protein, which can affect the body's glycolipid metabolism (22)], diglycerides [part of soybean oil, which can reduce visceral fat (23)], stachyose [part of soybean oligosaccharides, which can meet the needs of people with diseases (24)]. In addition, whether there are other subdivided components with physiological functions is to be further explored.

## 4. Conclusion

In short, the articles in this Research Topic only present a small portion of the latest research on all topics. Based on these topics and articles, this editorial suggests some potential directions for the high-value utilization of soybean nutrients, which may still limit. As an important plant-based functional food ingredient, the high-value utilization of soybean nutrients is still in its infancy and requires all researchers to continue to contribute their research work and wisdom.

## Author contributions

BL: writing—original editorial. FW and SQ: supervision. YL: visualization. HY: supervision and validation. All authors contributed to the article and approved the submitted version.
